# Pulmonary Vein Isolation in a Patient with Superior Vena Cava Atresia: Challenges and Solutions

**DOI:** 10.19102/icrm.2022.13123

**Published:** 2022-12-15

**Authors:** Mohsin Khan, Lakshmi Muthukumar, Imran Niazi

**Affiliations:** ^1^Aurora Cardiovascular and Thoracic Services, Aurora Sinai/Aurora St. Luke’s Medical Centers, University of Wisconsin School of Medicine and Public Health, Milwaukee, WI, USA

**Keywords:** Ablation, cryoballoon, left superior vena cava

## Abstract

Congenital cardiac anomalies pose a significant challenge during cardiac ablation procedures. Pre-procedural multimodality imaging can help to identify these incidental findings, which may assist with procedural planning to achieve successful outcomes. Here, we describe the technical challenges associated with cryoballoon ablation of the pulmonary veins in a patient with persistent left superior vena cava who was found to have right superior vena cava atresia during the case.

## Introduction

Persistent left superior vena cava (SVC) (PLSVC) occurs in approximately 0.2%–0.6% of the general population.^1^ There are significant anatomical variations associated with PLSVC, including atresia of the right SVC, dual SVC with and without anastomosis between the right and left SVCs, and PLSVC draining directly into the left atrium. PLSVC associated with right SVC atresia is extremely rare, occurring in 0.09%–0.13% of patients.^2^ We describe the case of a 55-year-old man with paroxysmal atrial fibrillation and known PLSVC and right SVC atresia who presented for cryoballoon ablation of the pulmonary veins (PVs). The procedural challenges encountered during the case will be discussed here, together with a review of the literature.

## Case presentation

**Figure 1:**
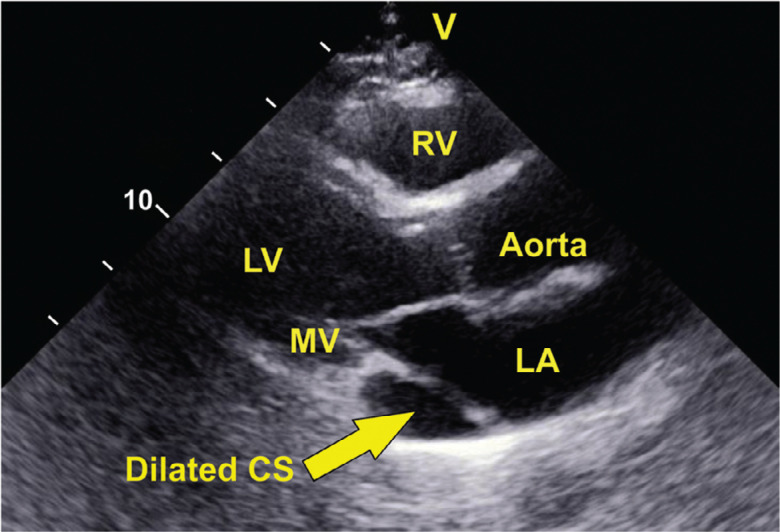
Transthoracic echocardiography in the parasternal long-axis view shows a dilated CS. *Abbreviations:* CS, coronary sinus; LA, left atrium; LV, left ventricle; MV, mitral valve; RV, right ventricle.

**Figure 2:**
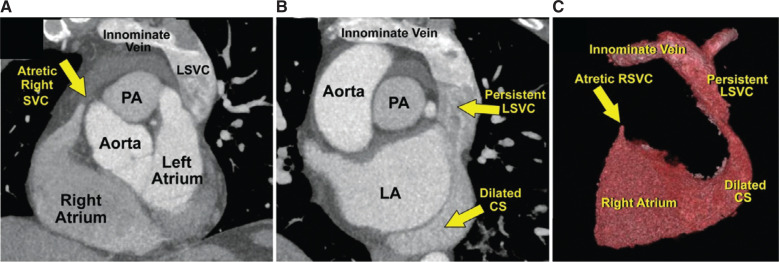
**A:** Computed tomography shows an atretic right superior vena cava (SVC) and left SVC connected to the innominate vein. **B:** Persistent left SVC draining into a dilated coronary sinus is seen. **C:** Three-dimensional reconstruction of a persistent left SVC and dilated coronary sinus is shown. *Abbreviations:* CS, coronary sinus; LA, left atrium; LSVC, left superior vena cava; PA, pulmonary artery; RSVC, right superior vena cava; SVC, superior vena cava.

**Figure 3:**
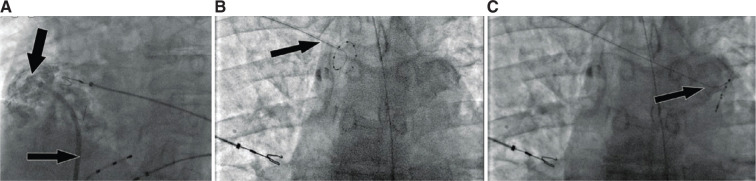
**A:** Intravenous contrast (large arrow) was injected through the transseptal sheath (small arrow) with no flow noted in the superior vena cava (SVC), confirming SVC atresia. **B:** High-output pacing with the spiral multipolar catheter (arrow) in the right subclavian vein without right phrenic nerve capture is seen. **C:** The spiral multipolar catheter (arrow) was advanced into the persistent left SVC with left phrenic nerve capture and high-output pacing.

A 55-year-old man with symptomatic paroxysmal atrial fibrillation despite being on sotalol was admitted for cryoballoon ablation of the PVs. Before the procedure, the patient underwent transthoracic echocardiography, which demonstrated a normal left ventricular ejection fraction and a dilated coronary sinus **(Figure 1)**. Computed tomography (CT) of the heart structure showed 2 right and 2 left PVs and a PLSVC with a significantly dilated coronary sinus **(Figure 2)**. In the electrophysiology laboratory, a diagnostic quadripolar catheter was advanced via the left femoral vein and positioned in the right atrium. Right SVC atresia was suspected when the quadripolar catheter could not be advanced into the SVC and was confirmed by contrast injection into the right atrium through the transseptal sheath **(Figure 3A)**. Although atresia of right SVC was not mentioned in the CT image of the heart structure, a retrospective review of the CT data post-procedure demonstrated an atretic right SVC **(Figure 2A)**. Because the right SVC was atretic, the transseptal sheath could not be advanced into the SVC and was gradually retracted to find the fossa ovalis in the usual way. A transseptal puncture was performed under intracardiac echocardiography (ICE) guidance. The fossa ovalis was visualized with the ICE catheter. The transseptal sheath was placed in the right atrium with the tip across the superior-most aspect of the tricuspid valve just above the His-bundle recording, then retracted gradually and rotated clockwise until the tip was observed under ICE guidance to lie in the mid-fossa ovalis. Then, it was slowly withdrawn until it came to lie in the anterior–inferior fossa ovalis, which is our preferred transseptal site for cryoballoon PV isolation. An NRG® RF Transseptal Needle (Baylis Medical, Mississauga, Ontario, Canada) was used for transseptal puncture under fluoroscopic and ICE guidance, and the transseptal puncture was completed without incident. Subsequently, the FlexCath Advance Steerable Sheath with an Arctic Front Advance cryoablation catheter and an Achieve spiral multipolar catheter (Medtronic, Minneapolis, MN, USA) inside it was advanced into the left atrium. The left-sided PVs were ablated using the double-freeze method.

Right phrenic nerve pacing prior to right-sided PV isolation presented challenges because the atretic right SVC prevented the usual retrograde approach to the phrenic nerve vicinity. Right axillary vein access was obtained to attempt right phrenic nerve pacing. High-output pacing (40 mA, 2 ms) in the right axillary vein was performed initially with a quadripolar catheter without success. This was replaced with a spiral multipolar catheter (Achieve) to ensure better contact with the vein walls, again without successful capture of the right phrenic nerve **(Figure 3B)**. The catheter was advanced through the innominate vein all the way to the left SVC with capture of the left phrenic nerve **(Figure 3C)**, but right phrenic nerve capture could not be obtained.

As cryoballoon isolation of the right-sided PVs was not considered safe without right phrenic nerve pacing, we considered using radiofrequency (RF) ablation for this purpose. Prior to RF ablation, PV potentials were assessed in the right PVs using the multipolar spiral catheter. No PV potentials were detected in either of the 2 veins. Therefore, no further ablation was performed.

The patient has been doing well and has had no further recurrences of atrial fibrillation over the last 2 years. No anti-arrhythmics or anticoagulants were given.

## Discussion

PLSVC is an important structure in the initiation and maintenance of atrial fibrillation.^3^ However, it is often discovered incidentally and can result in significant procedural challenges when encountered.^4^ Visualization of a very large coronary sinus on the echocardiogram should raise suspicion for a PLSVC.^5^ About 90% of PLSVCs connect to the right atrium via the coronary sinus, whereas 10% connect directly to the left atrium.^2^ Most patients with PLSVC also have right SVC with or without anastomosis.^6^ In the setting of an atretic right SVC, cryoballoon isolation of the right PVs presents special challenges.^7^ Right phrenic nerve pacing proximal to the ablation site is performed to avoid phrenic nerve palsy as this nerve courses adjacent to the origin of the right superior, and sometimes even the right inferior, PV. McKelvey and Chodosh and Santoro et al. each described a method of pacing the right phrenic nerve in this setting by placing a decapolar catheter through the coronary sinus and PLSVC–right SVC anastomosis. Both were successful in attaining phrenic nerve capture with this technique.^8,9^ We proceeded with direct right axillary vein access and paced along the length of the right subclavian vein utilizing both a quadripolar catheter and a spiral multipolar catheter without successful capture of the right diaphragm. We confirmed that the lack of right phrenic capture was not due to anesthetic agents or paralytics as we were able to achieve left phrenic capture. The aforementioned 2 case reports suggest that pacing from the right subclavian vein is a feasible option to capture the right diaphragm, but our case demonstrated that this is not always possible.

## Conclusions

Our case highlights the procedural considerations that need to be taken into account prior to proceeding with cryoballoon ablation in the rare instance of a patient with PLSVC and atretic right SVC. If a PLSVC is suspected based on a large coronary sinus on pre-procedural echo, a pre-procedural CT should be carefully reviewed for an atretic SVC with the radiology service. CARTO or other 3-dimensional mapping patches should be placed before the procedure to facilitate RF ablation of the right PVs if phrenic nerve capture is not possible. Right phrenic nerve pacing needs to be ensured prior to proceeding with cryoablation in this patient population. If phrenic nerve capture is not possible, RF-based wide area circumferential isolation of the PVs should be considered.
